# Postoperative pain in treatment of spontaneous pneumothorax with limited two-port thoracoscopy

**DOI:** 10.12669/pjms.304.5047

**Published:** 2014

**Authors:** Fengwu Lin, Chuan Zhang, Qiang Zhang, Dan Dang, Yan Zhao

**Affiliations:** 1 Fengwu Lin, Department of Thoracic Surgery, China-Japan Union Hospital of Jilin University, Changchun 130033, China.; 2 Chuan Zhang, Department of Pediatric Surgery, The First Hospital of Jilin University, Changchun 130021, China.; 3 Qiang Zhang, Department of Thoracic Surgery, China-Japan Union Hospital of Jilin University, Changchun 130033, China.; 4 Dan Dang, Department of Neonatology, The First Hospital of Jilin University, Changchun 130021, China.; 5 Yan Zhao, Department of Endocrine, The Second Hospital of Jilin University, Changchun 130041, China.

**Keywords:** Limited Two-Port Thoracoscopy, Spontaneous pneumothorax, Video assisted thoracic surgery

## Abstract

***Objectives:*** To investigate effects of the limited two-port video assisted thoracic surgery on treatment of spontaneous pneumothorax.

***Methods:*** A retrospective analysis of 96 patients with spontaneous pneumothorax who underwent video assisted thoracic surgery was conducted in the present study, in which 23 cases underwent the limited two-port video assisted thoracic surgery while 73 cases treated with the standard three-port video assisted thoracic surgery or the standard two-port video assisted thoracic surgery. The mean operation time, mean intraoperative blood loss and average postoperative hospital stay, average postoperative chest tube duration and postoperative pain rating were analyzed.

***Results:*** There was no statistical difference existed in the mean operation time, mean intraoperative blood loss, average postoperative hospital stay, average postoperative chest tube duration between the two groups (P>0.05). However, the postoperative pain in the limited two-port video assisted thoracic surgery group was significantly lower than that of the traditional video assisted thoracic surgery group.

***Conclusion:*** Compared with that of the standard three-port video assisted thoracic surgery or the standard two- port video assisted thoracic surgery, there is better cosmetic effect, and lower grade postoperative pain in the limited two-port video assisted thoracic surgery.

## INTRODUCTION

To date video assisted thoracic surgery (VATS) has become a main surgical method of treating spontaneous pneumothorax with a smaller incision and a faster recovery than other operation methods.^1^^,^^2^ Satisfactory treatment effects are achieved even in the treatments of some refractory spontaneous pneumothorax by the thoracoscopic operation.^3^ Generally, VATS operation often uses the double operating ports method or single operating port method termed as the standard three-port method^4^ or the standard two- port method.^5^

Recently, the VATS method for treating spontaneous pneumothorax has been improved by us, which is termed as the limited two-port method using an handle hole with a diameter of 1.5 cm smaller than the standard operating port. Postoperative pain and treatment effects of the limited two-port VATS were analyzed compared to the standard three-port or two-port VATS in our study.

The objective of the study was to investigate effects of the limited two-port video assisted thoracic surgery on treatment of spontaneous pneumothorax

## METHODS

***Patients: ***From August of 2010 to September of 2012, 96 patients with spontaneous pneumothorax were collected in this retrospective analysis, and they were all diagnosed by chest X-ray or chest Computer Tomography and treated by thoracoscopic operations in China-Japan Union Hospital of Jilin University. All enrolled patients were free of haematological diseases, disfunctions of heart, liver, spleen, kidney, stomach or intestine. Each patient signed an informed consent form. Approval was obtained from the institutional review committee of Jilin University. The patients were divided into two groups according to the surgical methods: 23 patients in the limited video assisted thoracic surgery (Group LVATS) underwent the limited two-port VATS and 73 patients in the traditional video assisted thoracic surgery (Group TVATS) treated by the traditional standard three-port or the standard two- port VATS.

**Fig.1:**
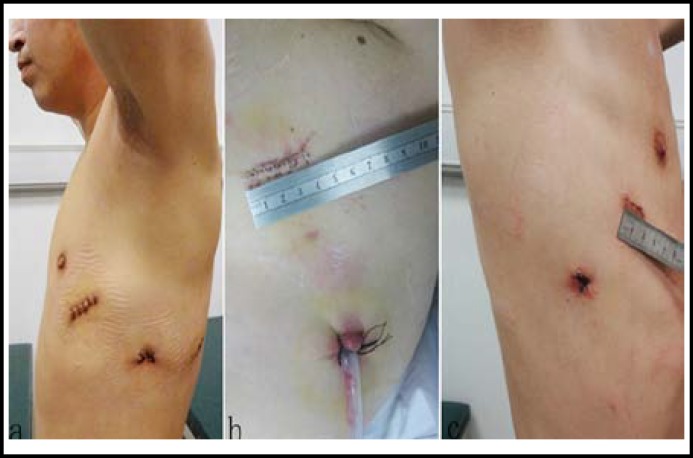
A handle hole with a diameter of 4.0-5.0 cm was made in the standard two-port or three port VATS method **(a, b),** a handle hole with a diameter of 1.5 cm in the limited two-port VATS surgery** (c).**

***Study design: ***All patients in the two groups were anaesthetized by the double lumen intubation method and they were placed in the maximally flexed lateral decubitus position tilted slightly backward to prevent the hip from obstructing downward movement. In the limited two-port VATS group, an incision with a length of about 1.5cm at the sixth or seventh interspace along mid-axillary line was used as the camera port in the limited two-port thoracoscopic group. An incision with a length of about 1.5cm at the fourth or fifth interspace between the midclavicular line and the anterior axillary line was made and used as the operating port (Fig.1). The position of bulla was detected carefully from apex of lung to base of lung during the operation. When the bulla position was confirmed, it was lifted by a slender shaft curve endoscopic forcep typically used in laparoscopic operations. In the meantime, lung tissues that were about 1cm away from bulla baseline were removed by a stapler. If possible, several stitches were used to consolidate the edge. During the process, a better operation angle was obtained by switching the camera port and the operating port. Pleural abrasion with the cotton balls was used to promote adhesion of pleura. When a lung inflation test was used to confirm that no air leak existed, chest was closed after indwelling the thoracic drainage tube at the camera port site.

Meanwhile, the traditional VATS group either employed the standard three-port VATS method during which an additional port was made at the posterior axillary line 6 or 7 intercostal space to assist operation or employed the standard two-port VATS method in which an operating port with a diameter of 4.0-5.0cm was made (Fig.1). During operation, bulla was detected and removed as described in the limited two-port group.

***Evaluation criteria of treatment effects: ***Evaluation criteria included operation time, postoperative treatment time, intraoperative bleeding, postoperative thoracic drainage tube duration of time and postoperative pain scores. The postoperative pain rating followed the pain grading method of the World Health Organization (WHO). Briefly, there was no pain in the 0 class. In the I class (mild pain), pain was tolerant, and it did not disturb sleeping or limit daily activity, and people could work. In the II class (middle pain), pain was obvious, and it disturbed sleeping, and people usually required general analgesic, sedative, hypnotic drugs. In the III class (severe), pain was acute with autonomic nerve functional disturbance, and it disturbed sleeping dramatically, and people usually required narcotic drugs.

***Statistical analysis: ***All measured parameters including mean operation time, average postoperative hospital stay, intraoperative bleeding and average postoperative chest tube duration were weighted, analyzed by the statistical software program Statistical Product and Service Solutions (SPSS) 17.0 (SPSS Inc., Chicago, IL, USA) and expressed as mean±standard deviation ( ±s), and t-test was used. Enumeration data including gender, prevalence frequency and postoperative pain score were analyzed by χ2 test. *P* <0.05 was considered significant.

## RESULTS

***Baseline characteristics: ***There were no significant difference (*P*>0.05) in general data including age, gender and primary or recurrence between the two groups (Table-I).

***Evaluation of treatment effects: ***Mean operation time, operative blood loss, average hospital stay after operation and average postoperative thoracic drainage tube duration of time were analyzed ,showing no statistical difference existed (*P*>0.05) (Table-II). Air leak occurred in 2 patients of the limited two-port group, and in 8 patients of the traditional thoracoscopic group. Both of them with postoperative air leak recovered without special treatment in 4 days. No hemothorax, empyema, atelectasis or other complications were found in both groups. Postoperative follow-up was conducted for 1-6 months without recurrence.

***Postoperative pain ratings: ***Postoperative pain ratings in all the postoperative patients were shown as below (Table-III). Five patient pain scores of the limited two-port VATS group were in the I or II class, while 43 of the traditional VATS group in the I or II class. 18 patient pain scores of the limited two-port VATS group were in the 0 class, while 30 of the traditional VATS group in the 0 class. The postoperative pain score in the limited two-port VATS group was significantly lower than that of the traditional VATS group (P<0.05).

## DISCUSSION

Primary spontaneous pneumothorax is one of common diseases in thoracic surgery, usually induced by ruptured bullae of lung. Treatment of primary spontaneous pneumothorax includes indwelling closed thoracic tube and operative treatment. While only indwelling thoracic drainage tube has a high relapse rate, better clinical effects are achieved in patients treated by operation.^6^^,^^7^ And to date operations of primary spontaneous pneumothorax typically adopt the VATS^8^^-^^10^, which has made tremendous progress in the past few decades. However in traditional standard three-port or two-port VATS with a diameter of 4.0-5.0 cm occurs a high risk of postoperative pain, only one handle hole with a diameter of 1.5 cm dose the limited two-port VATS have, which is adopted by us. Compared to the traditional VATS method in treating spontaneous pneumothorax disease, the limited two-port VATS could achieve the same clinical treatment effects, while with smaller incision and significantly lower grade postoperative pain.

**Table-I:** Baseline characteristics of study patients

***Characteristic***	***Group LVATS***	***Group TVATS***	***P value***
Age (year)	25±3.9	24±2.4	0.142
Gender			
Female	63	10	0.066
Male	16	7	
Primary/Recurrence	16/7	55/18	0.582

**Table-II:** Efficacy of the two groups

	***Group LVATS***	***Group TVATS***	***P value***
Mean opeation time (min)	50.0±4.1	51.4±4.0	0.327
Average hospital stay (d)	6.0±1.4	6.6±1.4	0.122
Average thoracic tub duration(d)	4.5±1.1	4.8±1.1	0.377

**Table-III:** Postoperative pain ratings

	***Group LVATS***	***Group TVATS***	* ***P value***
0	18	30	0.002
I~II	5	43	

* χ2=9.663

Though, there were some reports^11^^,^^12^ on treatment of spontaneous pneumothorax by the single port VATS with a relatively larger operating port of a diameter 4.0-5.0cm, the diameter of operating port in the limited two-port operation was less than 2.0 cm, and the injury of muscles and nevers was smaller. Postoperative pain in the limited VATS group was significantly lower than that of the traditional group (P<0.05) in this study. Meanwhile, no significant difference existed between the two groups according to the analysis of mean operation time, operative blood loss, average hospital stay after operation, occurrence rate of air leak after operation. Effectively, many complicated moves can be done by using instruments such as a slender shaft curve endoscopic forcep typically used in laparoscopic operations, and by switching the handle hole and camera hole.

This current study has provided some important implications for the clinical treatment of spontaneous pneumothorax. Firstly, reduced postoperative pain in the limited two-port VATS benefits those senile patients. Many clinical practices have shown that although the clinical treatment effects of the aged have been improved in treating by thoracoscopic operation^13^, the common complications of lung infection after operation were often induced by pre-operative pulmonary diseases such as COPD, and expectoration difficulty is obvious. Reduced postoperative pain helps cough and expectoration, promoting the patients’ recovery. And since no handle hole on back was adopted in the limited two-port operation, nursing staff can button back to assist patients to sputum and avoid airway secretions deposition after the operation. In turn all of these were helpful for recovery. In additon due to the single operating port with a diameter of about 1.5 cm locating below breast, the limited two-port thoracoscopic operation can meet the beauty requirements of young female patients. The naturally dropped breast can cover cuts. The camera port and the operating port can be located at the same level in designing, and the dressed loose bra can completely cover the cuts. Without affecting the operation effects, the small and secret cuts were achieved. In addition, if the scope with a diameter of 5.0 mm was used, the diameter of the camera port was only 6.0 mm, which was more cosmetic.

Conclusionly, the limited two-port thoracoscopic operation used in the our study attained better cosmetic effect, and lower grade postoperative pain, and has practical implications for patients, especially for senile patients and young female patients. Undoubtedly, the limited two-port VATS merits the attention of thoracic surgeons as a new alternative treatment for spontaneous pneumothorax in patients.

## Notes

***Note: ***This research received no specific grant from any funding agency in the public, commercial or not-for-profit sectors.
